# Dynamics of the soil respiration response to soil reclamation in a coastal wetland

**DOI:** 10.1038/s41598-021-82376-0

**Published:** 2021-02-03

**Authors:** Xiliang Song, Yihao Zhu, Weifeng Chen

**Affiliations:** 1 College of Resources and Environment, Shandong Agricultural University, Tai’an, 271018 China; 2 Shandong Provincial Engineering and Technology Research Center for Phyto-Microremediation in Saline-Alkali Land, Shandong, China

**Keywords:** Climate sciences, Ecology, Environmental sciences, Solid Earth sciences

## Abstract

The soil carbon (C) pools in coastal wetlands are known as “blue C” and have been damaged extensively owing to climate change and land reclamation. Because soil respiration (RS) is the primary mechanism through which soil carbon is released into the atmosphere at a global scale, investigating the dynamic characteristics of the soil respiration rate in reclaimed coastal wetlands is necessary to understand its important role in maintaining the global C cycle. In the present study, seasonal and diurnal changes in soil respiration were monitored in one bare wetland (CK) and two reclaimed wetlands (CT, a cotton monoculture pattern, and WM, a wheat–maize continuous cropping pattern) in the Yellow River Delta. At the diurnal scale, the RS at the three study sites displayed single-peak curves, with the lowest values occurring at midnight (00:00 a.m.) and the highest values occurring at midday (12:00 a.m.). At the seasonal scale, the mean diurnal RS of the CK, CT and WM in April was 0.24, 0.26 and 0.79 μmol CO_2_ m^−2^ s^−1^, and it increased to a peak in August for these areas. Bare wetland conversion to croplands significantly elevated the soil organic carbon (SOC) pool. The magnitude of the RS was significantly different at the three sites, and the yearly total amounts of CO_2_ efflux were 375, 513 and 944 g CO_2_·m^−2^ for the CK, CT and WM, respectively. At the three study sites, the surface soil temperature had a significant and positive relationship to the RS at both the diurnal and seasonal scales, and it accounted for 20–52% of the seasonal variation in the daytime RS. The soil water content showed a significant but negative relationship to the RS on diurnal scale only at the CK site, while it significantly increased with the RS on seasonal scale at all study sites. Although the RS showed a noticeable relationship to the combination of soil temperature and water content, the synergic effects of these two environment factors were not much higher than the individual effects. In addition, the correlation analysis showed that the RS was also influenced by the soil physico-chemical properties and that the soil total nitrogen had a closer positive relationship to the RS than the other nutrients, indicating that the soil nitrogen content plays a more important role in promoting carbon loss.

## Introduction

The soil organic carbon (SOC) pool, which undergoes a dynamic and direct exchange with the atmospheric C pool, plays an important role in the global carbon (C) cycle^1^. Changes in SOC stocks even at a small amplitude can reportedly result in intense impacts on atmospheric carbon dioxide (CO_2_) concentrations^2^. Although wetlands constitute no more than 7% of the earth's land, 225 Pg (billion tons) of C, accounting for one-fourth of the global SOC, has been reserved in them^3^. Coastal wetland is a primary wetland type. The soil C pool in coastal wetlands, which remains in a relatively stable state known as “blue C”, plays an important role in maintaining the balance of the global C cycle^4^. Currently, climate change and soil degradation due to anthropogenic activity have caused 1/3 of the coastal wetland degeneration, resulting in 0.15–1.02 Pg of CO_2_ emissions from the soil to the atmosphere each year^5^. With increased global warming and human activity, much more attention should be paid to the C sequestration of coastal wetlands^4,6^.

Long-term experimental studies have confirmed that SOC is highly sensitive to changes in land-use patterns which from native ecosystems (e.g., wetland, forest and grassland ecosystems) to agricultural systems, resulting in dramatic SOC losses^7^. The impacts of land-use changes on physical and chemical soil properties (e.g., the total nitrogen contents, available phosphorous contents, soil pH, and bulk densities) in different ecosystems have been presented in several articles^8,9^. Tidal wetlands (native salt marshes) reclaimed for use as urban development zones, pasture lands and croplands showed significantly altered organic C storage capacity and reduced soil C pools^10^. Zhang et al.^11^ also found a remarkable decrease in SOC after natural wetlands were converted to grazed pastures or agricultural fields. However, the influence of soil reclamation and agricultural activities (such as fertilizer and irrigation applications, and crop cultivation) accompanying the conversion from wetlands to croplands on soil C dynamics cannot be distinguished clearly because soil C dynamics can be significantly affected by land use. Moreover, the effects of land-use changes on C sequestration in wetlands belonging to estuaries or deltas are still unknown.

The primary path through which soil C is released into the atmosphere is soil respiration (RS)^12^. The environmental changes that increase the RS even over a small amplitude may significantly enhance the atmospheric CO_2_ levels. The CO_2_ releases from the soil to the atmosphere^13^ will inevitable exacerbate the global climate warming^14^. On the global scale, RS can release approximately 50–98 Pg C annually into the atmosphere^15,16^. Furthermore, RS has been shown to be a sensitive indicator of overall soil metabolic activities and can be used to determine the degree of soil recovery in restored ecosystems^17^. Thus, studying the RS changes in various ecosystems is important for predicting the possible C feedback between terrestrial and atmospheric ecosystems and for improving C cycle models.

Accurately estimating RS changes in natural and reclaimed wetlands (farmland) and determining their limiting factors play important roles in predicting the future global C equilibrium and its possible climate change consequences. The Yellow River Delta is the newest coastal wetland in the world and is not only one of the most active land–ocean interaction areas worldwide^18^ but is also a potential C sink, and anthropogenic activity has been shown to be a key factor in SOC change^3^. With its rapid economic development and dramatic land-use changes, the natural wetlands in the Yellow River Delta are experiencing dramatic degradation^19^, and many natural wetlands have been reclaimed as croplands. By the studies of Scott et al.^20^ and Shi et al.^21^, the change in land use not only altered the vegetation community structure but also markedly influenced both soil microbiological and physico-chemical characteristics, thereby resulted in RS change. Although a few studies have been undertaken to explore changes in soil C sequestration in the reclaimed wetlands of the Yellow River Delta^22,23^, few studies have focused on the effects of reclamation activities on the seasonal and diurnal RS or the factors controlling RS in coastal wetlands to date. Therefore, the coastal wetland in the Yellow River Delta was chosen to be the target study site, and the diurnal and seasonal changes in the RS rate in a bare wetland and two reclaimed wetlands (cotton mono-cropping, and winter wheat and summer maize rotation cropping) were measured in situ with the closed dynamic chamber method. The objectives of this study were (1) to determine the diurnal and seasonal variation in soil CO_2_ efflux during the growing and non-growing seasons in a wetland and two neighbouring farmlands and (2) to elucidate the mechanisms of environmental factors (such as the soil temperature, soil water content, land use types and soil physico-chemical properties) affecting the RS.

## Results

### Soil temperature and water content dynamics

**Figure 1:**
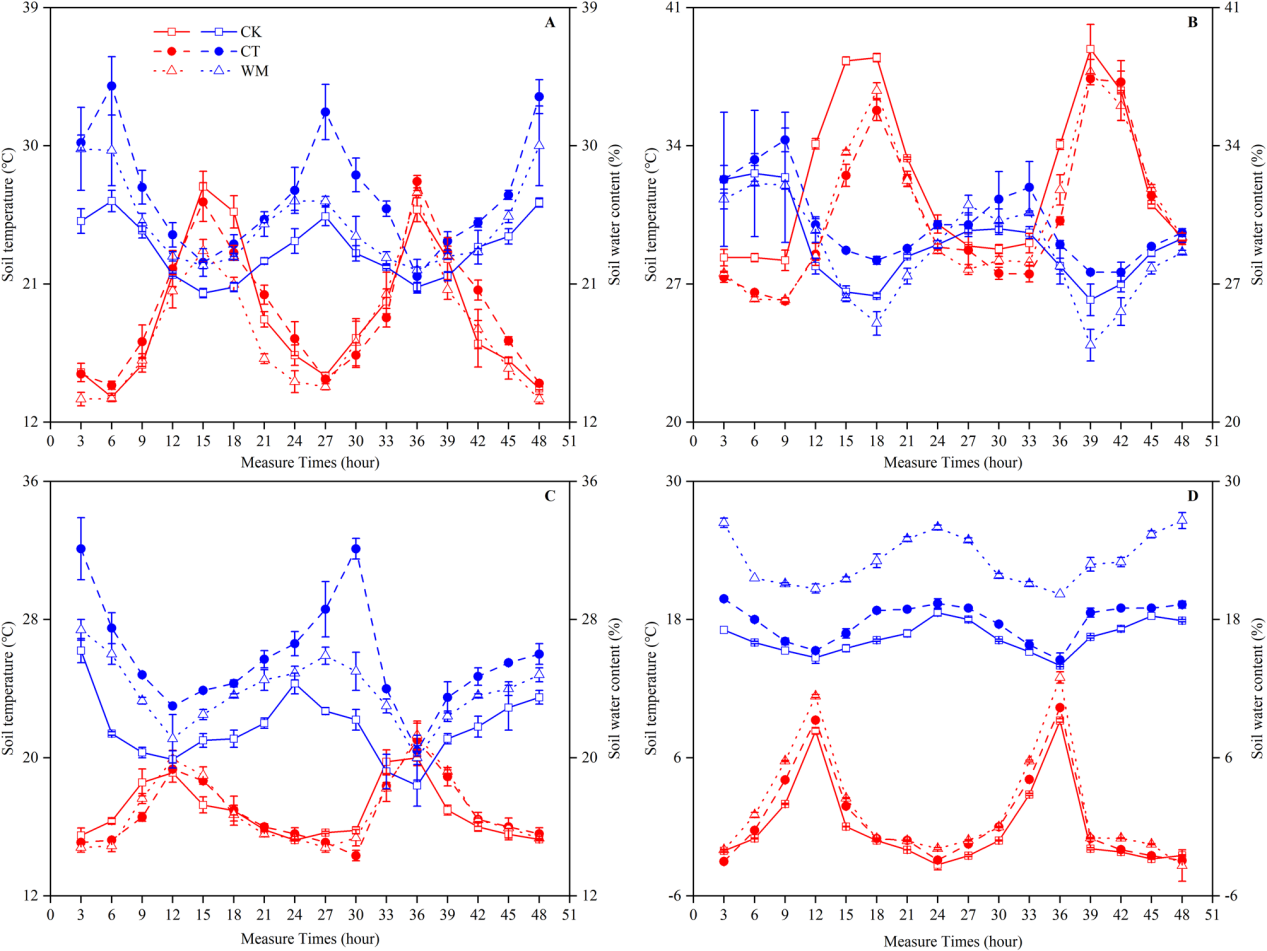
Dynamics of the soil temperature (red colour) and soil water content (blue colour) at a 0–10 cm soil depth in April (**A**), August (**B**), October (**C**) and December (**D**) at the study sites. CK, natural coastal wetland; CT, cotton mono-cropping farmland; WM, wheat–maize rotation farmland. Vertical bars represent ± SD of the mean (n = 3).

The changes in the soil temperature and water content at different study sites over 48 h are shown in Fig. 1. On the diurnal scale, the lowest soil temperature values for the three study sites were in the middle of the night (0:00–3:00 a.m.), whereas the highest values occurred at midday (12:00 a.m.). The diurnal dynamics of the soil temperatures in April, August, October and December exhibited similar trends, such that the soil at all the sites in the summer (August) had a higher temperature (26.1–33.7 °C), and in the winter (December), it had a lower temperature (− 2.0–13.0 °C). The marked difference in the soil temperatures at the different sites were shown for August, and the temperature in the CK was higher than the temperature in the CT and WM because there was no vegetation covering the soil surface in the CK. In contrast, changes in the diurnal soil water contents showed the opposite trend for soil temperature. The lowest soil water contents at the three study sites occurred at midday (12:00 a.m.), whereas the highest values were found in the middle of the night (0:00–3:00 a.m.). On an annual scale, the average soil water contents of the CK, CT and WM were 23.2, 26.9% and 25.0% in April, 29.0, 30.1 and 28.7% in August; 21.8, 25.8 and 23.9% in October; and 16.5, 17.9 and 23.2% in December, respectively.

### Changes of soil physico-chemical properties

The physical and chemical soil properties of the reclaimed sites (CT and WM) and un-reclaimed site (CK) measured at the end of each campaign are provided in Table 1. The bulk density (BD) for all the treatments had a stable value throughout the different seasons, but the CK value (1.64–1.65 g·cm^−3^) was higher than that of the CT (1.44–1.47 g·cm^−3^) or WM (1.40–1.44 g·cm^−3^). Bare soil had very high soluble salts (SS) that varied during different months. During cold months (April and December), the SS values in the CK were 12.57 and 13.22‰, respectively. However, in the warm months (August and October), the SS values in the CK decreased by one-quarter. After decades of reclamation, the SS in the CT and WM significantly decreased, to 1.67–3.63‰, with high values in April and December and low values in August and October, respectively. The pH values at all the sites were not significantly different and maintained a relatively stable value (7.56–8.10) throughout the different months. During the same season, both the soil organic carbon (SOC) and soil microbial carbon (MBC) at the reclaimed sites (CT and WM) were higher than those at the un-reclaimed sites (CK). The change in the SOC was not significant in different months, and the MBC showed the highest values (89.20–117.23 mg·kg^−1^) in August and the lowest values (46.53–49.00 mg·kg^−1^) in December. The levels of soil nutrients, e.g., total nitrogen (TN), available phosphorus (AP) and available potassium (AK) in the CK, were much lower than those of the CT and WM and exhibited temporal heterogeneity.

**Table 1:** Changes in the soil physical and chemical properties of different study sites at the 0–10 cm depth in April, August, October and December.

Parameters	April			August			October			December		
	CK	CT	WM	CK	CT	WM	CK	CT	WM	CK	CT	WM
BD (g·cm^-3^)	1.65 ± 0.01a	1.47 ± 0.03b	1.44 ± 0.03b	1.65 ± 0.01a	1.44 ± 0.02b	1.42 ± 0.03b	1.64 ± 0.07a	1.47 ± 0.03b	1.44 ± 0.03b	1.65 ± 0.01a	1.46 ± 0.02b	1.40 ± 0.03c
SS (‰)	12.57 ± 0.18a	3.63 ± 0.86b	2.56 ± 0.21b	8.06 ± 0.44a	2.37 ± 0.37b	1.67 ± 0.52b	9.91 ± 0.31a	2.24 ± 0.37b	1.97 ± 0.18b	13.22 ± 0.02a	2.24 ± 0.01c	2.65 ± 0.23b
pH	7.61 ± 0.24a	7.59 ± 0.25a	7.56 ± 0.06a	7.91 ± 0.08a	8.01 ± 0.01a	8.10 ± 0.01a	7.83 ± 0.05a	7.73 ± 0.10a	7.88 ± 0.06a	7.73 ± 0.09a	7.86 ± 0.08a	7.69 ± 0.01a
SOC (g·kg^−1^)	6.57 ± 0.15c	10.29 ± 0.05b	12.03 ± 0.38a	6.43 ± 0.19c	11.17 ± 0.40b	14.33 ± 0.17a	6.50 ± 0.08c	9.53 ± 0.40b	10.40 ± 0.33a	7.13 ± 0.21c	12.43 ± 0.12b	14.30 ± 0.16a
MBC (mg·kg^−1^)	44.47 ± 2.36b	52.13 ± 4.84a	54.53 ± 3.33a	89.20 ± 4.78a	90.87 ± 3.07a	117.23 ± 6.98a	57.73 ± 0.35b	63.00 ± 4.68b	88.97 ± 7.01a	46.53 ± 5.73a	47.53 ± 5.89a	49.00 ± 2.52a
TN (g·kg^−1^)	1.66 ± 0.17b	2.82 ± 0.20a	2.94 ± 0.20a	1.65 ± 0.14c	4.12 ± 0.06b	6.40 ± 0.04a	0.53 ± 0.08b	1.23 ± 0.19a	0.80 ± 0.15b	0.60 ± 0.06b	0.80 ± 0.32b	1.12 ± 0.17a
AP (mg·kg^−1^)	11.44 ± 0.42b	26.53 ± 1.75a	27.15 ± 1.51a	8.75 ± 0.15b	11.55 ± 0.06a	11.40 ± 0.27a	7.20 ± 0.15b	11.03 ± 0.66a	11.42 ± 0.19a	8.12 ± 0.48c	5.36 ± 0.80b	11.93 ± 0.33a
AK (mg·kg^−1^)	209.3 ± 0.41c	313.7 ± 0.16a	283.5 ± 1.47b	118.6 ± 1.96	240.83 ± 1.76a	232.45 ± 1.67b	176.5 ± 1.68c	330.40 ± 4.17a	301.47 ± 8.82b	223.75 ± 0.61c	265.43 ± 4.54b	349.57 ± 6.56a

K, natural coastal wetland; CT, cotton mono-cropping farmland; WM, wheat–maize rotation cropping farmland; BD, bulk density; SS, soluble salts; SOC, soil organic carbon; MBC, microbial biomass carbon; TN, total nitrogen; AP, available phosphorus; and AK, available potassium.

Values are represented as means ± SD. Different letters in the same column indicate significant variance by T-test at *p* < 0.05.

### Dynamics of soil respiration

**Figure 2:**
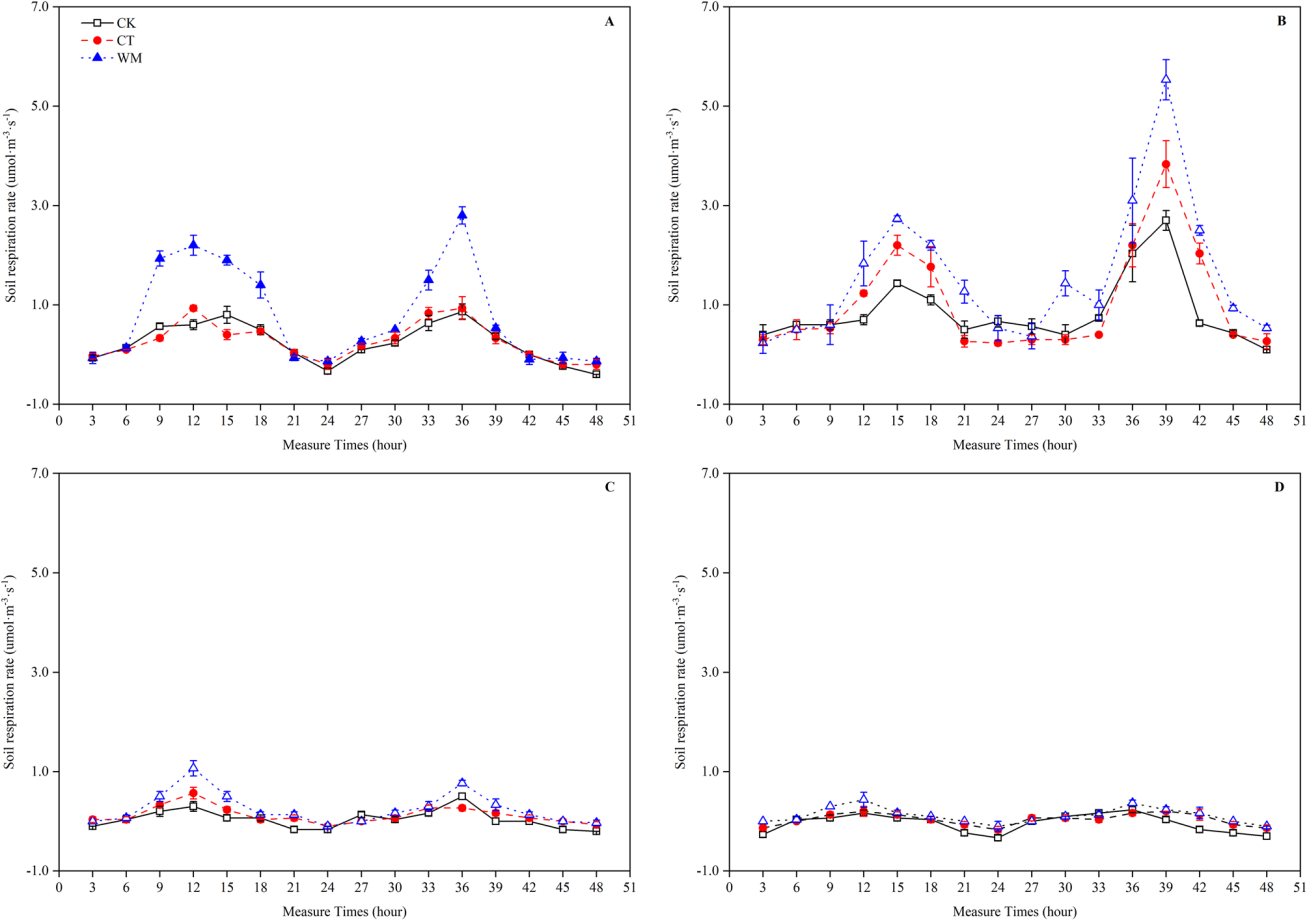
Diurnal dynamics of soil respiration in April (**A**), August (**B**), October (**C**) and December (**D**) of 2017. CK, natural coastal wetland; CT, cotton mono-cropping farmland; WM, wheat–maize rotation farmland. Vertical bars represent ± SD of the mean (n = 3).

The diurnal and seasonal changes in the RS at different study sites are shown in Fig. 2. At a seasonal scale, there were significant differences during various months. In the spring, the average RS of the CK, CT and WM was 0.24, 0.26 and 0.79 μmol CO_2_·m^−2^·s^−1^, respectively. In the summer, the average RS of the CK, CT and WM was 0.85, 1.05 and 1.58 μmol CO_2_·m^−2^·s^−1^, respectively. In autumn, the average RS of the CK, CT and WM was 0.04, 0.12 and 0.25 μmol CO_2_·m^−2^·s^−1^, respectively. In winter, the average RS of the CK, CT and WNT was − 0.04, 0.04 and 0.11 μmol CO_2_·m^−2^·s^−1^ respectively.

At the diurnal scale, the RS during different seasons was represented by single-peak curves for the CK, CT and WM (Fig. 2). The RS in the CK during the 48 measured hours in the spring, summer, autumn and winter was − 0.40–0.87 μmol CO_2_·m^−2^·s^−1^, 0.40–2.03 μmol CO_2_·m^−2^·s^−1^, − 0.20–0.50 μmol CO_2_·m^−2^·s^−1^ and − 0.33–0.23 μmol CO_2_·m^−2^·s^−1^, respectively. The RS in the CT during the 48 measured hours in the spring, summer, autumn and winter was − 0.20–0.93 μmol CO_2_·m^−2^·s^−1^, 0.27–2.20 μmol CO_2_·m^−2^·s^−1^, − 0.10–0.27 μmol CO_2_·m^−2^·s^−1^ and − 0.17–0.20 μmol CO_2_·m^−2^·s^−1^, respectively. The RS in the WM during the 48 measured hours in the spring, summer, autumn and winter was − 0.13–2.80 μmol CO_2_·m^−2^·s^−1^, 0.23–3.10 μmol CO_2_·m^−2^·s^−1^, − 0.10–0.77 μmol CO_2_·m^−2^·s^−1^ and − 0.10–0.23 μmol CO_2_·m^−2^·s^−1^, respectively. Furthermore, the highest and lowest RS values for all the study sites occurred at midday (with measuring times at 12 and 36 h) and midnight (with measuring times at 24 and 48 h) each day.

The annual cumulative RS at the different study sites in the Yellow River Delta differed. Land reclamation activities greatly enhanced the annual cumulative RS. Compared to that of the CK (375 g CO_2_·m^−2^), the annual cumulative RS of the CT and CK significantly increased, by 25.6% and 103.6%, respectively.

### Relationship between diurnal soil respiration and soil temperature and water content

**Figure 3:**
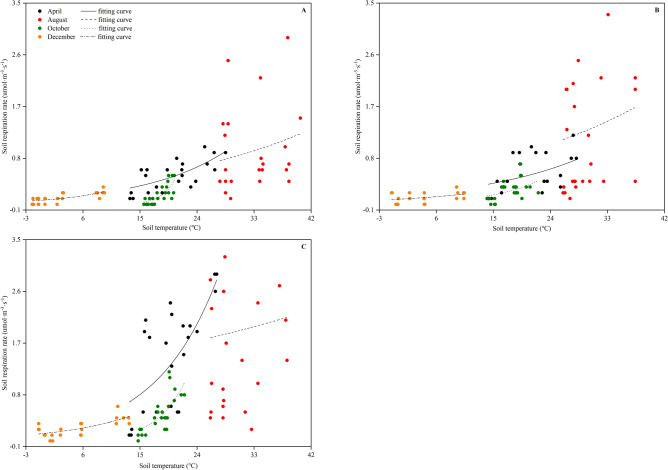
Relationships of the daytime soil respiration in April, August, October and December to the soil temperature at the CK **(A)**, CT **(B)** and WM **(C)** sites. Different types of lines are the nonlinear fits for different months. CK, natural coastal wetland; CT, cotton mono-cropping farmland; and WM, wheat–maize rotation cropping farmland.

The relationships between the RS of the CK, CT and WM and the soil temperature on the diurnal scale are provided in Fig. 3, and the fitted exponent regression equations are shown in Table 2. The RS of the CK, CT and WM during different months (except for August) showed a significant positive relationship (*p* < 0.01) to soil temperature at a soil depth of 10 cm (Fig. 3). At the CK site, the soil temperatures in April, October, and December accounted for 50, 52 and 28% of the variation in RS values, respectively (Table 2). At the CT site, the soil temperatures during these three seasons accounted for 18, 41 and 20% of the variation in the RS, respectively (Table 2). At the WM site, the soil temperatures during these three seasons accounted for 46, 51 and 51% of the variation in the RS, respectively (Table 2). The calculated temperature sensitivity (Q_10_) of the RS in the CK, CT and WM is provided in Table 2. The values of Q_10_ showed significantly difference at the three sites (*p* < 0.05). In April, the Q_10_ of the CK, CT, and WM was 2.2, 1.8, and 2.8, respectively. In October, the Q_10_ of the CK, CT, and WM had a higher value, 3.5, 6.3, and 7.6, respectively. In December, the Q_10_ of CK, CT, and WM decreased to 2.0, 1.8, and 2.4, respectively.

**Table 2:** Correlations of the diurnal soil daytime respiration with the soil temperature and soil moisture.

Month	Treatment	Fitting function	Q10	R^2^	*P*
April	CK	RS = 2.674–0.095 × W	2.2 ± 0.1e	0.47	< 0.01
		RS = 0.009 × e^0.078×T^		0.50	< 0.01
		Ln(RS) = − 0.840 + 0.013 × T + 0.296 × W − 0.014 × W × T		0.51	< 0.01
	CT	RS = 1.890–0.053 × W	1.8 ± 0.0f	0.37	< 0.01
		RS = 0.155 × e^0.058×T^		0.18	< 0.01
		Ln(RS) = 6.430–0.221 × T − 0.083 × W + 0.001 × W × T		0.31	< 0.01
	WM	RS = 6.660–0.215 × W	2.8 ± 0.1d	0.34	< 0.01
		RS = 0.172 × e^0.103×T^		0.46	< 0.01
		Ln(RS) = − 0.289–0.041 × T + 0.129 × W – 0.002 × W × T		0.41	< 0.01
October	CK	RS = 2.303–0.105 × W	3.5 ± 0.3c	0.61	< 0.01
		RS = 0.023 × e^0.129×T^		0.52	< 0.01
		Ln(RS) = 163.055–8.072 × T − 7.816 × W + 0.383 × W × T		0.63	< 0.01
	CT	RS = 0.966–0.029 × W	6.3 ± 0.1b	0.31	< 0.01
		RS = 0.008 × e^0.184×T^		0.41	< 0.01
		Ln(RS) = 45.3–1.859 × T − 2.194 × W + 0.087 × W × T		0.37	< 0.01
	WM	RS = 3.360–0.126 × W	7.6 ± 0.1a	0.58	< 0.01
		RS = 0.006 × e^0.203×T^		0.51	< 0.01
		Ln(RS) = 29.171–1.430 × T − 1.333 × W + 0.065 × W × T		0.55	< 0.01
December	CK	RS = 1.331–0.079 × W	2.0 ± 0.2f	0.56	< 0.01
		RS = 0.094 × e^0.069×T^		0.28	< 0.01
		Ln(RS) = 8.045–0.677 × T − 0.426 × W + 0.030 × W × T		0.55	< 0.01
	CT	RS = 0.626–0.031 × W	1.8 ± 0.01f	0.13	< 0.05
		RS = 0.108 × e^0.061×T^		0.20	< 0.01
		Ln(RS) = − 8.028–0.329 × T + 0.895 × W− 0.051 × W × T		0.10	< 0.01
	WM	RS = 2.206–0.093 × W	2.4 ± 0.1e	0.22	< 0.05
		RS = 0.131 × e^0.089×T^		0.51	< 0.01
		Ln(RS) = − 12.169–0.462 × T + 0.166 × W − 0.001 × W × T		0.53	< 0.01

RS, soil respiration; T, soil temperature; W, soil water content; CK, natural coastal wetland; CT, cotton mono-cropping farmland; and WM, wheat–maize rotation cropping farmland.

Different letters in the same column indicate significant variance by T-test at *p* < 0.05.

**Figure 4:**
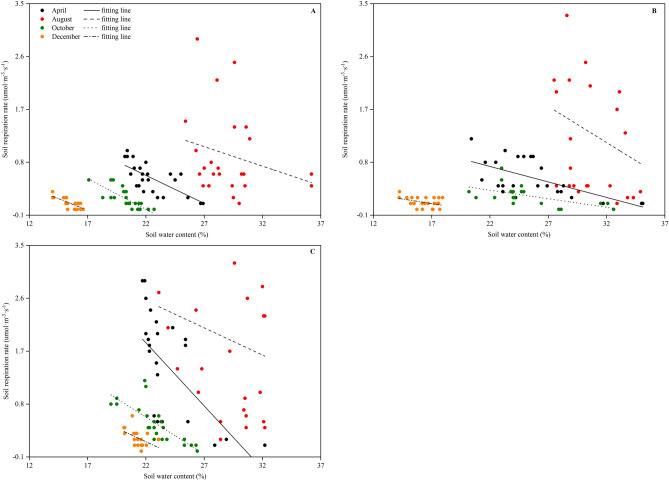
Relationships of daytime soil respiration in April, August, October and December with soil water contents at the CK **(A)**, CT **(B)** and WM **(C)** sites. Different types of lines are the linear fits for different months. CK, natural coastal wetland; CT, cotton mono-cropping farmland; and WM, wheat–maize rotation cropping farmland.

The relationships between the RS of the CK, CT and WM to the soil water content and the fitted linear equations on the diurnal scale are shown in Fig. 4 and Table 2, respectively. In contrast to the relationship between the RS and soil temperature in Fig. 3, the RS of the CK, CT and WM for different months (except August) showed a significant negative relationship (*p* < 0.05) to the soil water content at a soil depth of 10 cm (Fig. 4). At the CK site, the soil temperatures in April, October, and December accounted for 47, 61 and 56% of the variation in the RS, respectively (Table 2). At the CT site, the soil temperatures of these three seasons explained 37, 31 and 13% of the variations in the RS, respectively (Table 2). At the WM site, the soil temperature from these three seasons accounted for 34, 58 and 22% of the variation in the RS, respectively (Table 2).

An empirical model using both the soil temperature and water content as independent variables was fit to the data set with a simultaneous measurement of the RS, soil temperature and water content to reflect the interactive effect of both factors (Table 2). The combination of the soil temperature and water content at the diurnal scale showed a significant effect (*p* < 0.01) on the RS at all three study sites. At the CK site, the combined soil temperature and water content in April, October, and December accounted for 51, 63 and 55% of the variation in the RS, respectively (Table 2). At the CT site, the soil temperature and water content of these three seasons explained 31, 37 and 10% of the variation in the RS, respectively (Table 2). At the WM site, the soil temperature and water content during these three seasons accounted for 41, 55 and 53% of the variation in the RS, respectively (Table 2).

### Relationship between annual soil respiration and soil temperature and water content

**Figure 5:**
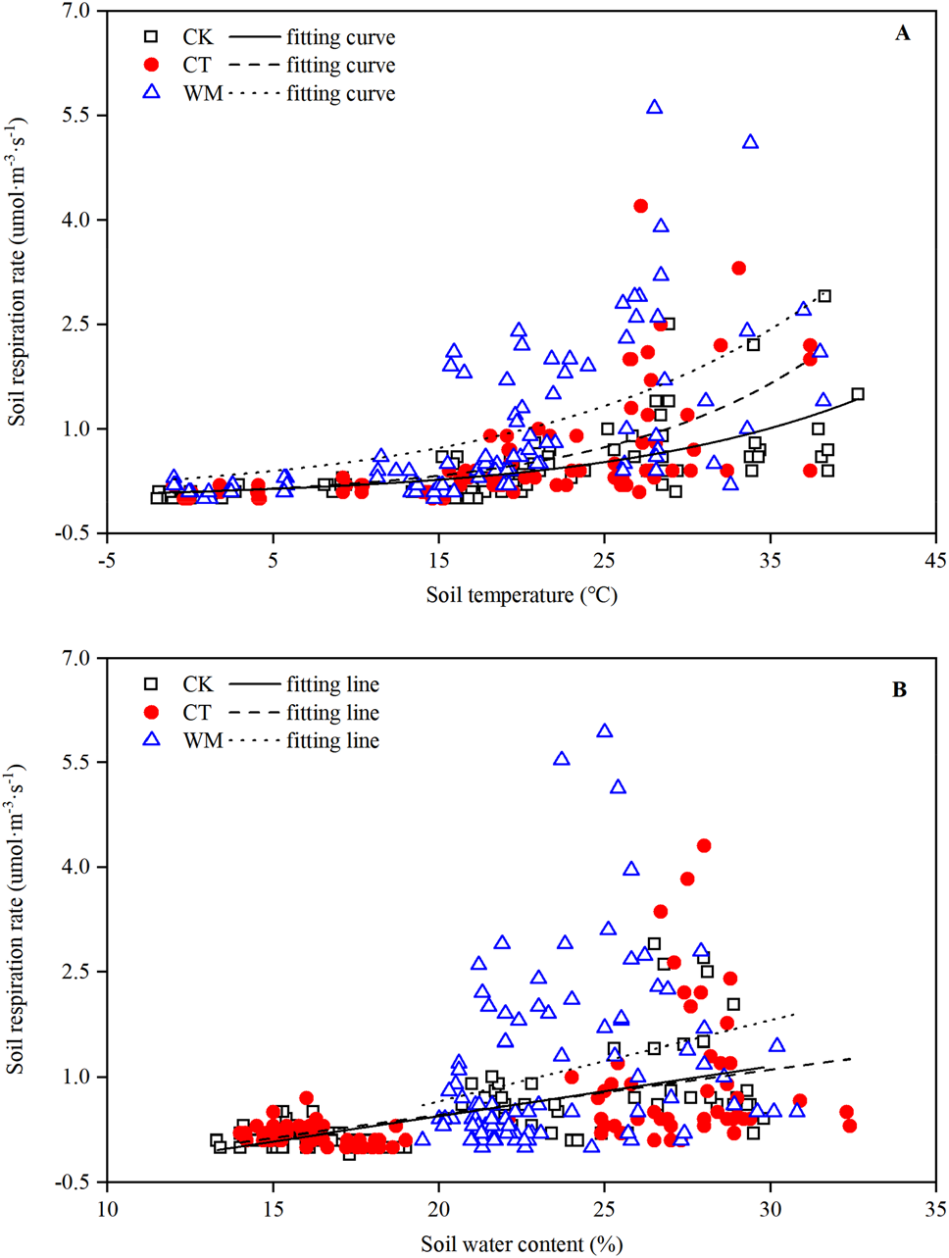
Relationships of daytime soil respiration to the soil temperature (**A**) and water content (**B**). Different types of lines are the linear fits in different months. CK, natural coastal wetland; CT, cotton mono-cropping farmland; and WM, wheat–maize rotation cropping farmland.

The relationships between the RS of the CK, CT and WM and the soil temperature and water content on an annual scale are provided in Fig. 5 A and B, and the fitted exponent regression equations are shown in Table 3. The RS in the CK, CT and WM showed a significant positive nonlinear relationship to the soil temperature (Fig. 5 A, *p* < 0.01) and a noticeable positive linear relationship to the soil water content (Fig. 5 B, *p* < 0.01). At the CK site, the soil temperature and water content accounted for 42 and 47% of the variation in the RS, respectively. At the CT site, the soil temperature and water content accounted for 35 and 22% of the variation in the RS, respectively. At the WM site, the soil temperature and water content accounted for 33 and 6% of the variation in the RS, respectively. The synergic effects of the soil temperature and water content on the RS of the CK, CT and WM were also remarkable, explaining 44, 43 and 41% of the variation in the RS, respectively (**Table ****3**).

**Table 3:** Correlations of seasonal soil daytime respiration with the soil temperature and soil moisture.

Treatment	Fitting function	Q_10_	R^2^	*P*
CK	RS = − 0.988 + 0.071 × W	1.9 ± 0.1b	0.37	< 0.01
	RS = 0.103 × e^0.065×T^		0.42	< 0.01
	Ln(RS) = − 3.441 + 0.073 × T + 0.049 × W − 0.0103 × W × T		0.44	< 0.01
CT	RS = − 0.835 + 0.065 × W	2.2 ± 0.0a	0.22	< 0.01
	RS = 0.100 × e^0.080×T^		0.35	< 0.01
	Ln(RS) = − 1.270–0.028 × T − 0.110 × W + 0.007 × W × T		0.43	< 0.01
WM	RS = − 1.653–0.001 × W	1.8 ± 0.0c	0.06	< 0.05
	RS = 0.297 × e^0.060×T^		0.33	< 0.01
	Ln(RS) = − 0.268–0.035 × T − 0.085 × W-0.006 × W × T		0.41	< 0.01

RS, soil respiration; T, , soil temperature; and W, soil water content.

Different letters in the same column indicate significant variance by T-test at *p* < 0.05.

### Relationship between soil respiration and soil physico-chemical properties

**Figure 6:**
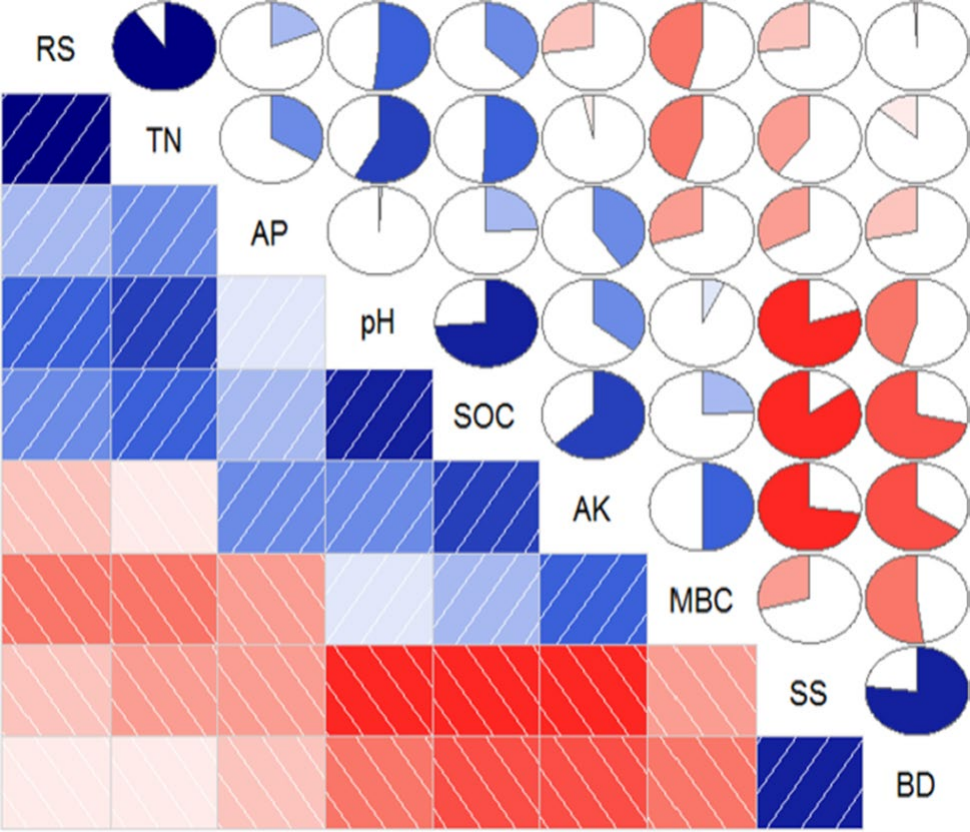
Correlation analysis between the soil respiration and soil physico-chemical properties. Red rectangles and circles indicate a negative relationship between different properties; blue rectangles and circles indicate a positive relationship between different properties; the area of the colour in the circle indicates the correlation coefficient, and the darker red or blue colours indicate a closer relationship. RS, soil respiration; BD, bulk density; SS, soluble salts; SOC, soil organic carbon; MBC, microbial biomass carbon; TN, total nitrogen; AP, available phosphorus; and AK, available potassium.

The correlation analysis between the RS and soil physico-chemical properties is shown in Fig. 6. RS had a positive relationship to soil total nitrogen, available phosphorus, pH, and soil organic carbon, while the relationship between the RS and the other soil properties, such as available potassium, microbial biomass carbon, soluble salts, and bulk density, was negative. Based on the calculation of the correlation coefficient, the strongest relationship was found between RS and changes in the soil total nitrogen, as indicated by the high value of the coefficient (0.91). The correlation coefficients of the RS with the other soil physico-chemical properties were lower than 0.50.

## Discussion

In our study, the annual cumulative RS in the CT and WM of the Yellow River Delta was 513 and 944 g CO_2_·m^−2^, which was within the range of the RS (160–1418 g CO_2_·m^−2^) for global cropland^24^ and higher than the CK (375 g CO_2_·m^−2^) by 25.6% and 103.6%, respectively. The results indicate that bare wetland soil reclaimed as cropland caused a great deal of C loss, and the C loss in the wheat–maize rotation cropping field was much higher than that of the cotton monoculture field. Similar findings were also presented in Iost et al.^25^ and Bu et al.^26^. In addition, although the diurnal RS values in the reclaimed wetlands were higher than those of the bare lands, and the wheat–maize continuous cropping farmland was higher than the cotton mono-cropping farmland in the crop grown season (Fig. 2), these findings do not indicate that the reclaimed wetland ecosystem caused greater C loss. The reason is that plants have a strong net C assimilation ability through photosynthesis, and the cover crops of this farmland have a higher carbon sequestration ability than vegetation in bare soil, which compensates for the soil C loss by net C assimilation from the cover^27^. In this context, whether the reclaimed wetlands cause a C loss or not depends on the balance of plant photosynthesis and RS, and further studies over the entire Yellow River Delta ecosystem are needed in the future.

Notably, the average RS of the CK during night in April, October and December was − 0.05, − 0.07 and − 0.19 μmol CO_2_·m^−2^·s^−1^, respectively (Fig. 2). Although the average RS values of the CT and WM at night for those months were higher than those of CK, there were still some RS values below 0 μmol CO_2_·m^−2^·s^−1^, especially at 24:00 p.m. Similar results were also found for the RS in the *Phragmites australis* and *Tamarix chinensis* communities in the Yellow River delta wetland during autumn and winter^28^. In fact, the biochemical process of RS is catalysed by the enzyme activity in the soil, and the temperature is the first and primary limiting factor affecting this enzyme activity^29^. When the soil temperature is below 0 °C, the RS is very low because of the weak metabolic rates of the roots and microbes under cold weather conditions. However, the negative value of the RS in our results seems impossible, and the low soil temperature cannot explain this phenomenon completely because the soil temperature at night in April and October exceeded 10 °C in our study (Fig. 1). The saline and alkaline soil at the study sites might explain this phenomenon. According to Xie et al.^30^, CO_2_ is absorbed at a rate of 0.3–3.0 μmol CO_2_·m^−2^·s^−1^ at the interface between soil water and ambient air by alkaline soil through an inorganic, non-biological process. Then, some of the atmospheric CO_2_ can be dissolved into the soil water to form carbonic acid (H_2_CO_3_) to partially neutralize the alkalinity of the soil water in saline/alkaline soils. When the CO_2_ absorbed by the soil exceeds the CO_2_ emissions from the root and microbes, the measured RS was negative.

It is generally accepted that the RS increases with increasing soil temperature^31^, and the annual RS is closely related to the average annual temperatures^32^. The diurnal changes in the RS in the CK, CT and WM all consistently exhibited an inverted “U” curve pattern from 0:00 a.m. to 24 p.m. in April, August, October and December, with maximum values being found at 12:00 a.m. and minimum values appearing at 24:00 p.m. (Fig. 2). This result was different from that of Wang et al.^4^ who showed that the RS in the Yellow River Delta peaked at 12:00 a.m. and was lowest at 6:00 a.m. and that of Liu et al.^33^ who showed that the highest RS value on Taihang Mountain was found at 13:00–15:00 p.m. and the lowest value occurred at 6:00–8:00 a.m. These different studies may indicate that the change in the RS coincides with the diurnal change in the soil temperature (Fig. 1). A similar finding was also reported by Wang et al.^4^. At the seasonal scale, with the increase in soil temperature, the RS started to increase in April, reached its highest value in August, and then declined to a low value after November, which is consistent with the findings of Li et al.^34^. The seasonal variation in the diurnal RS rate shown in Fig. 2 also showed that the soil temperature was the primary factor affecting the RS rate in the Yellow River Delta^4^. Elevated temperatures increased the RS because warming might increase soil biological activity and the decomposition of the SOC and litter^35^. The correlation coefficients of the RS to the soil temperatures in the CK, CT and WM for different months are shown in Table 3. At all the study sites, the soil temperature had a significant relationship to the RS in April, October and December, but the relationship in August was not significant (*p* < 0.01), suggesting that the soil temperature was not the limiting factor for RS during the warm season. This finding can be explained by temperature acclimation during the high growing season^36^, and compared with that of thermophilic crops (e.g., cotton), the RS for cold-resistant crops (e.g., wheat) is more sensitive to temperature changes^37^. The results indicated that the soil temperature at a low range is the primary limiting factor for RS, while the temperature under conditions suitable for other environmental factors may play a more important role in RS^38^.

The temperature sensitivity of the RS (as expressed by the Q_10_) is widely used to indicate the feedback between the global C cycle and climate change^39^. It should be noted that the non-linear curves in Fig. 3 were fitted with daytime RS values but not with night-time data at different temperatures because the RS at night was negative and cannot be simulated. In April, October and December, the RS values of the CK, CT and WM increased with the temperature increase, and the relationship between the RS and the soil temperature were positive and significant (*p* < 0.01). However, there was no potential relationship between the RS and the soil temperature in August neither in the bare wetlands nor in the reclaimed wetlands (Fig. 3). This result supported the correlation coefficients of the RS to the soil temperatures in Table 2. The significant difference in the Q_10_ values from the three study sites (Tables 2 and 3) suggested that the temperature sensitivity of the RS is markedly influenced by the crop type. Similar results were also found by Jiang et al.^38^. Furthermore, the Q_10_ for the WM was higher than that for the CK and CT during different seasons, supporting the evidence showing that Q_10_ does not always have a constant value but tends to increase with decreasing soil temperature^40^, and the C sequestration in the winter wheat and summer maize rotation cropping farmland is more vulnerable than the cotton mono-cropping farmland and natural wetlands in future climate change scenarios.

**Figure 7:**
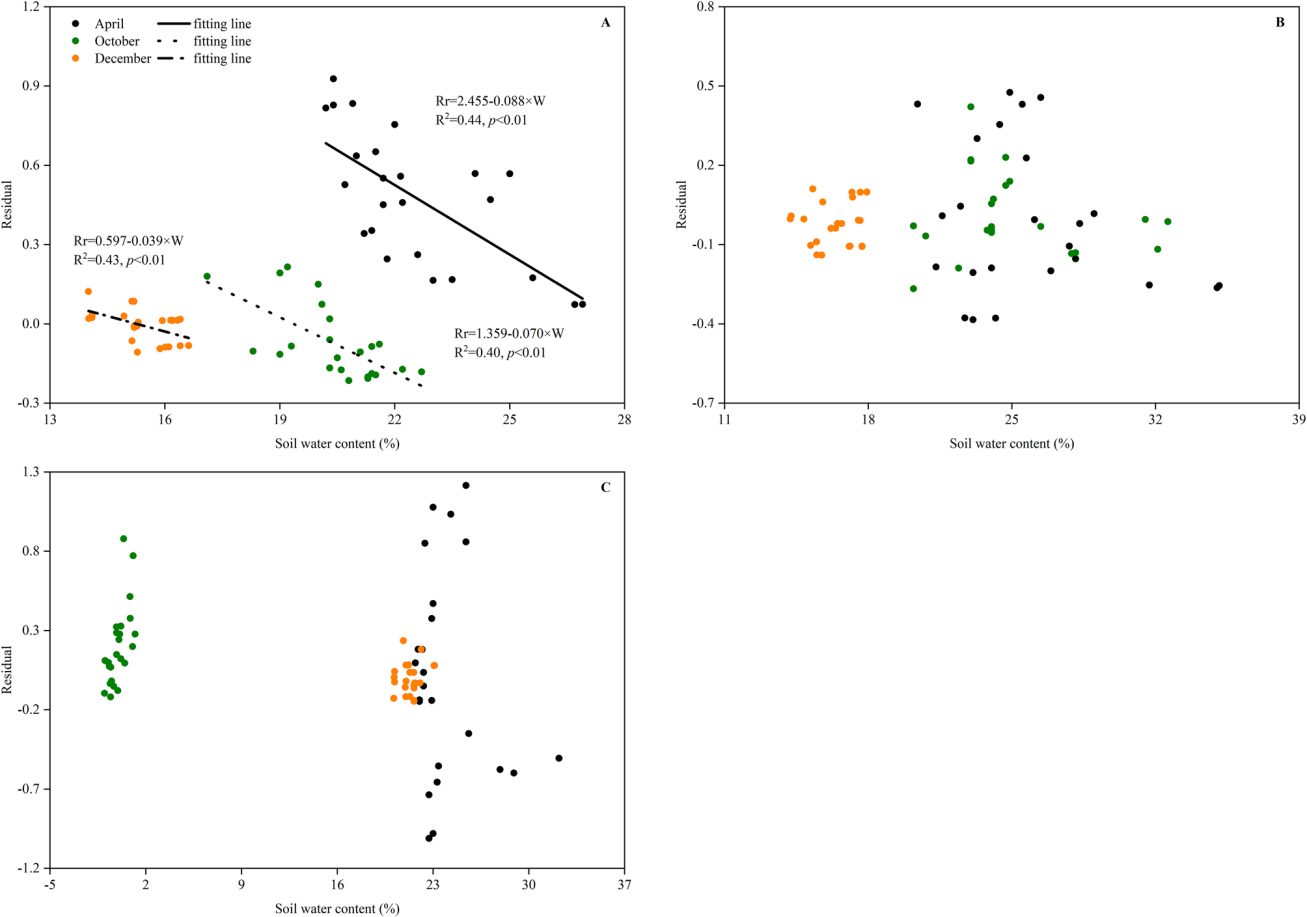
Relationships of daytime soil respiration residual in April, October and December with soil water contents at the CK **(A)**, CT **(B)** and WM **(C)** sites. Different types of lines are the linear fits for different months. The residual values of observed soil respiration and modeled values from the Q_10_ function were related to soil water content at 10 cm. CK, natural coastal wetland; CT, cotton mono-cropping farmland; and WM, wheat–maize rotation cropping farmland.

In addition to the temperature, soil respiratory processes have been reported in numerous studies to be strongly influenced by soil moisture, with drier soils tending to exhibit less CO_2_ efflux^41,42^. The temperature was reportedly the dominant factor, but the soil water content was the limiting factor that influenced the RS dynamics^43,44^. Other studies showed that the RS was highly correlated with changes in soil temperature only when water was not limited at a seasonal time scale^45,46^. Our study showed similar results: the RS of the CK, CT and WM consistently exhibited significant linear correlations (*p* < 0.05) with the soil water content at both the diurnal (Fig. 4 and Table 2) and seasonal scales (Fig. 5B and Table 3), indicating that the soil water content strongly affected the CO_2_ emissions from both natural and reclaimed coastal wetlands. At the seasonal scale, the RS at the three study sites showed significant linear positive correlations with the soil water content. Similar findings were also found by Guo et al. (2019)^47^ and Zhang et al. (2010)^48^. However, the relationship between the RS and the soil water content at the diurnal scale was significantly negative (Fig. 4 and Table 2), suggesting that the soil water content inhibited CO_2_ efflux at a small temporal scale. The result contradicts that from most other studies^42,49^. One possible reason is that the low groundwater depth (ranging from 0.6 m to 2.4 m) and the high groundwater mineralization degree (ranging from 6.3 g·L^−1^ to 18.6 g·L^−1^) in the study area increased the salt content of the soil water, resulting in strong soil salt stress on the normal metabolism of the plant roots and microbes^1,4^, which was confirmed by the negative correlation between the RS and the SS content in Fig. 6. What’s more, the remarkable negative relationships between soil temperature and soil water content (Fig. 1) potentially suggested that the close relationships between RS and soil water content on diurnal scale (Fig. 4) may be spuriously. To separate the confounding effects of soil temperature and water on RS, residuals between observed values of RS and those from the Q_10_ function^50^ were used to build the relationship with soil water content (Fig. 7). After subtracting the temperature-dependent RS, the residuals exhibited a noticeable negative linear correlation (*p* < 0.01) with the soil water content at the CK site (Fig. 7A) while their relationships at the CT and WM sites (Fig. 7B and C) were not significantly (*p* > 0.05). The similar results in Figs. 7A and 4A suggested the CO_2_ emissions from natural coastal wetland was strongly influenced by the soil water content on diurnal scale. On the other hand, the observed results in Fig. 7B and C which were contradictory to Fig. 4B and C indicated that the soil water content was not the limiting factor on RS on diurnal scale in reclaimed coastal wetlands. Furthermore, the significant difference between natural and reclaimed coastal wetlands reflected human activities’ strong effects on the improvement of soil water availability in reclaimed coastal wetlands^51,52^.

Although the soil temperature and water content were considered two alternative factors that affected the temporal dynamics of the RS in the present study, the effect of these two environment factors should not be studied separately when analysing in situ data. The reason is that their combined impact is more complex than the sum of their individual effects, especially in environments with high variability^53^. A two-variable empirical model was used in this study to reflect the combined effects of the soil temperature and water content on the RS. According to the fitted empirical equations in Tables 2 and 3, our findings indicated that the soil temperature and water content combination had a significant impact on the RS (*p* < 0.01). However, at the seasonal scale the percentages of the variance that were explained by the two-variable models were 44% at the CK site, 43% at the CT site, and 41% at the WM site (Table 3), indicating that the soil temperature and water content may not be the dominant influential factors in the study and that there are still some possible factors that impact the RS^54^. Schütt et al.^55^ found that, although the temperature and water content at the soil surface are the primary factors restricting RS, especially in low temperature environments, other environmental factors (such as low SOC and high soil salt) become the limiting factors when the temperature is suitable for root and microbe respiration. Similar findings were also reported by Zhang et al.^56^. In fact, the SOC content has been shown to be the primary factor in the stimulation of microbial respiration^57^, and the RS is unrelated to the temperature when the soil C content is lower than 9%^58^. In addition, the high level of soil salt is also one of the primary reasons for the reduced RS^59^, which is confirmed by the fact that the RS has a significant negative relationship with the soluble salt contents in soil^4^. In the present study, all the physical and chemical soil properties, including the bulk density, soluble salts, soil organic carbon, microbial biomass carbon, total nitrogen, available phosphorus and available potassium, varied by season and different land use type (Table 1), indicating that the RS variations can be explained by their combined effect^33^. According to the correlation analysis between the RS and the soil physico-chemical properties, shown in Fig. 5, the highest correlation coefficient (0.91) of the RS to the soil total nitrogen indicated that the soil total nitrogen is the primary limiting factor on the RS. Similar results were supported by Hu et al.^1^ that an increase in total nitrogen is a more important factor than salinity, which benefits the reproduction of β-proteobacteria and enhances the abundance of *Anaerolineae*, clearly enhancing heterotrophic soil microbial activities, soil microbial respiration and RS. The different results can be attributed to differences in the soil texture and physico-chemical properties, vegetation type, environmental conditions, and other factors^34,55,60^.

## Conclusion

In conclusion, both the diurnal and seasonal dynamics of the soil CO_2_ efflux for a natural wetland and two reclaimed wetlands used for farmland exhibited great variation. The natural wetland in the Yellow River Delta that was converted to cropland showed a significantly increased CO_2_ efflux and elevated SOC pool. The surface soil temperature had a significant positive relationship to the RS at both the diurnal and seasonal scales. The soil water content showed a significant but negative relationship to the RS at the diurnal scale only at the CK site, while significantly increasing the RS at the seasonal scale at all study sites. Although temporal RS showed a noticeable correlation with soil temperature and water content combination, the synergic effects of these two environment factors were not much greater than their individual effects. In addition, the RS was also influenced by the soil physico-chemical properties, and the soil nitrogen content played a more important role than other nutrients in promoting carbon loss.

## Methods

### Study area description

The field experiments were undertaken on April, August, October and December, 2017 on Bohai Farm (37°47′N, 118°36′E), which is located in Dongying Province, China. The climate at the study site is a northern subtropical marine monsoon climate, with clear distinctions between the four seasons over one year. Its mean annual evaporation, mean annual precipitation and mean annual temperature are approximately 1982 mm, 552 mm and 12.0 °C, respectively. The soil in the study site was classified as coastal saline fluvo-aquic soil with clay-loam texture^61^.

Based on the present and former land uses, three study sites including a bare wetland (CK) and two reclaimed farmlands (CT, a cotton monoculture pattern, and WM, a wheat–maize continuous cropping pattern) with three replicates were chosen within the research area. There was approximately 1000 m of distance between the reclaimed lands and the natural coastal wetland. The normal soil properties at the different study sites are shown in Table 1. The groundwater depth and groundwater mineralization degree in the study area were 1.8–2.0 m and 12.1–14.3 g/L in April, 0.5–1.2 m and 6.3–8.7 g·L^−1^ in August, 1.1–1.6 m and 13.2–16.8 g·L^−1^ in October, and 1.6–2.4 m and 15.3–18.6 g·L^−1^ in December, respectively.

Tillage was performed in both CT and WM before crop seeds were sown. Winter wheat (*Triticum aestivum* L., cv. Jimai 22) was planted at a seeding rate of 200 kg·ha^−1^ in October 2016 and harvested in June 2017 from 9 to 10 cm wide rows. Summer maize (*Zea mays* L., cv. Denghai, 605) was planted at 75,000 seeds·ha^−1^ in June 2017 and harvested in October 2017 from 60 to 70 cm wide rows. Cotton (*Gossypium hirsutum*, cv. Lumianyan 28) was planted at 30,000 seeds·ha^−1^ in April 2017 and harvested in October 2017 from 60 to 65 cm wide rows.

### Soil respiration measurement

The RS was measured in 23 April, 21 August, 17 October and 5 December of 2017 at the three experiment sites (CK, CT and WM) using a portable automated infrared soil CO_2_ flux system (LI-8100A, LI-COR Inc., Lincoln, USA). Three PVC collars, each with a diameter of 20 cm and a height of 15 cm were inserted into the soil, with 10 cm above the ground, before the RS measurements were taken at each site. The collars were left in place throughout the entire study period in the reclaimed lands, and they were installed one day before the measurements in the salt marshes to avoid tidal inundations. Each collar was installed at least 10 m away from one another. Before the measurements were taken, all visible living bodies (aboveground plants and litter) in the PVC collar were clipped and removed without disturbing the surface soil approximately two days before each field measurement campaign. Each measurement ran from 00:00 a.m. to 24:00 p.m. on a sunny day, and the frequency of the measurements was three hours. All the RS measurements were constant for two days at each time.

### Soil temperature and water content measurement

When the RS measurements were conducted, the soil temperature and water contents were recorded simultaneously at a 10 cm depth near each PVC collar using a Delta-T soil temperature and water sensor (WET-2-K1, Delta-t, Cambridge, England).

### Soil physicochemical properties measurement

After the RS measurements at the selected sites were concluded, surface soil samples (0–20 cm) were placed in polyethylene boxes and taken to the laboratory. After air-drying at room temperature for at least two weeks, the field-moist soil samples were sieved through a 2-mm nylon sieve to remove the plant roots, coarse debris and sand. The soil bulk density at the soil depth of 0–10 cm was measured using a cutting ring (5 cm in both diameter and depth).

The soil pH was determined in a 1:5 (w/v) soil: water slurry using a pH meter (Sartorius PB-10, Sartorius, Germany). The soluble salt (SS) content was determined by gravimetric method. The microbial biomass carbon (MBC) was determined using the fumigation-extraction method^62^. The SOC was analysed by potassium dichromate oxidation titration^63^. The total nitrogen (TN) content was measured using an Elemental Analyser (CHNOS Elemental Analyzer, Vario EL, Elementar, Germany). The available phosphorus and available potassium were measured using the Olsen method and flame emission spectrometry, respectively.

### Data analysis

#### Calculation of annual soil CO_2_ efflux

The annual soil CO_2_ efflux was calculated using the following model^64^:

1 $${\text{RS = }}\sum {\text{RS}}_{{\text{i}}} \times 3600\;{\text{s}}/{\text{h}}$$

where RS (μmol CO_2_·m^−2^·s^−1^) refers to the soil respiration rate; RS_i_ is the estimated rate of RS at hour i; s represents seconds; and h represents hours.

#### Relationship between the RS and the soil temperature

An exponential function was used to simulate the relationship between the RS (μmol CO_2_·m^−2^·s^−1^) and the soil temperature (°C):

2 $${\text{RS}} = {\text{ae}}^{{{\text{bT}}}}$$

where RS (μmol CO_2_·m^−2^·s^−1^) refers to the soil respiration rate; T (°C) refers to the soil temperature at a 10 cm soil depth; and a and b are two regression coefficients.

#### Q_10_ calculation

The Q_10_ value was calculated as follows:

3 $${\text{Q}}_{10} = {\text{e}}^{{10{\text{b}}}}$$

where b is obtained from Eq. (2).

#### Relationship between the RS and the soil water content

The relationship between the RS (μmol CO_2_·m^−2^·s^−1^) and the soil water content (W, % v/v) was analysed using a linear model^65^:

4 $${\text{RS}} = {\text{c}} + {\text{dW}}$$

where RS (μmol CO_2_·m^−2^·s^−1^) refers to the soil respiration rate; W (% v/v) refers to the soil water content at a 10 cm soil depth; and c and d are two linear regression coefficients.

#### Relationship between the RS and the combined soil temperature and water content

The relationship between the RS and the soil temperature and water content combination was analysed using an empirical model^66,67^:

5 $${\text{Ln}}\left( {{\text{RS}}} \right) = {\text{e}} + {\text{fT}} + {\text{gW}} + {\text{hTW}}$$

where RS (μmol CO_2_·m^−2^·s^−1^) refers to the soil respiration rate; T (°C) refers to the soil temperature at a 10 cm soil depth; W (% v/v) refers to the soil water content at a 10 cm soil depth; and e, f, g, and h are four regression coefficients.

#### Statistical analysis

All the data in the study are reported as the mean values with the standard deviation (± SD). All the statistics were performed using SPSS 19.0 (SPSS for Windows, Chicago, IL, USA). All the statistical tests were considered to be significant if *p* < 0.05, and a Duncan pairwise analysis was employed to analyse the significant differences. The correlations between the RS and soil physico-chemical properties were analysed with R language software (version 3.5.1). The graphs except that in Fig. 6 were created using Origin 9.0 software (Origin Lab, Massachusetts, USA).
